# The impact of different clinicopathologic factors and salvage therapies on cervical cancer patients with isolated para-aortic lymph node recurrence

**DOI:** 10.1007/s12672-023-00825-w

**Published:** 2024-03-01

**Authors:** Chenyan Fang, Yinfeng Zhu, Ping Zhang, Tao Zhu, Yingli Zhang

**Affiliations:** Zhejiang Cancer Hospital, Hangzhou Institute of Medicine (HIM), Chinese Academy of Sciences, Hangzhou, 310022 Zhejiang China

**Keywords:** Cervical cancer, Isolated para-aortic lymph node recurrence, Salvage therapy, Prognosis

## Abstract

**Background:**

Cervical cancer patients with isolated para-aortic lymph nodes (PALN) recurrence were mainly associated with treatment failure. For these patients, radiotherapy, chemotherapy, surgery ± adjuvant therapy or chemoradiotherapy may be advised, however, no specific therapy has been proposed yet. This study aimed to explore factors influencing the prognosis of cervical cancer cases with isolated PALN recurrence and to find out an effective salvage therapy.

**Methods:**

Cervical cancer cases with isolated PALN recurrence who received therapies in Zhejiang Cancer Hospital between January 2013 and June 2021 were analyzed retrospectively.

**Results:**

Carcinoembryonic antigen (CEA) level > 10 ng/mL and positron emission tomography/computed tomography (PET/CT) imaging method used to detect the recurrence were found to be associated with the local control rate. PALN (positive), squamous-cell carcinoma-antigen (SCC-Ag) level (> 10 ng/mL) upon initial diagnosis, and CEA level (> 10 ng/mL), number of metastatic lymph nodes (several) at recurrence were associated with worse survival. Compared with surgery ± adjuvant therapy, chemotherapy (CT) alone or sequential chemoradiotherapy (SCRT) was associated with worse PFS or OS. Concurrent chemoradiotherapy (CCRT) after PALN recurrence could reduce the risk of the second recurrence. 3-year OS of cases after surgery ± adjuvant therapy was the highest (65%), followed by CCRT (45.7%), SCRT (38.9%), radiotherapy (RT) (33.3%), and CT (20.6%).

**Conclusion:**

In cervical cancer patients with isolated PALN recurrence, chemoradiotherapy or surgery ± adjuvant therapy may be preferred as the salvage treatment.

**Supplementary Information:**

The online version contains supplementary material available at 10.1007/s12672-023-00825-w.

## Background

Cervical cancer is a common gynecologic malignancy. Although the majority of cervical cancer cases have high tumor control rates and a good prognosis after radical hysterectomy, definitive radiotherapy with or without concurrent chemotherapy, approximately 20–40% of cases develop recurrence [1, 2]. Para-aortic lymph nodes are the most common site of recurrence other than the pelvic cavity [3]. Isolated para-aortic lymph node (PALN) recurrence was defined as PALN was the only site of recurrence, with an incidence of approximately 1.7–12% [4]. Cases with isolated PALN recurrence were always associated with treatment failure after salvage therapies [5]. For such cases, radiotherapy, chemotherapy, surgery ± adjuvant therapy or chemoradiotherapy can be selected, as no specific therapy has been proposed yet.

Some retrospective studies have demonstrated that concurrent chemoradiotherapy could be regarded as an effective treatment modality [6–10]. Several studies have shown that squamous-cell carcinoma antigen (SCC-Ag) level, carcinoembryonic antigen (CEA) level, age at recurrence, the number of PALN recurrences, the highest level of PALN recurrence, size of the largest PALN recurrence, clinical symptoms at recurrence, time to PALN recurrence, and type of therapy after PALN recurrence were factors associated with survival [11–16].

These studies were all retrospective and their sample size was limited. Now we performed a retrospective analysis of this issue again in our center to access the outcome of cervical cancer patients with isolated PALNs recurrence and explore factors that may influence the prognosis. In order to provide some help in the selection of therapy type and improve the prognosis of these cases.

## Methods

### Patients

50 cervical cancer cases with isolated PALNs recurrence who received therapies in Zhejiang Cancer Hospital (Hangzhou, China) between January 2013 and December 2021 were retrospectively analyzed. These patients were histologically proven squamous cell carcinoma, adenocarcinoma and adenosquamous carcinoma of the cervix undergoing surgery with or without PALN dissection, radiation therapy, or concurrent chemoradiotherapy (CCRT) at initial diagnosis. Patients who had metastatic disease in addition to PALN, persistent disease after primary therapy and patients with incomplete radiotherapy were excluded in our study.

The study was approved by the Medical Ethics Committee of Zhejiang Cancer Hospital. Written informed consent was waived because of retrospective nature of the current study. All methods were performed in accordance with the relevant guidelines and regulations. We retrospectively collected data from medical records and out-patient/ telephone interviews, including characteristics of recurrence, clinicopathological features, age, treatment after isolated PALN recurrence, follow-up data, etc.

PALN recurrence was defined as enlarged PALN (> 10 mm diameter of the short axis) by computed tomography (CT) scan, magnetic resonance imaging (MRI), or PALN with abnormal uptake value on ^18^F-fluorodeoxyglucose positron emission tomography-CT (^18^F-FDG PET-CT) that were considered recurrence by radiologist.

### Salvage treatment (radiotherapy)

Radiotherapy was performed with 6-MV or 10-MV photons using linear accelerators.

The majority of cases received intensity-modulated radiation therapy (IMRT), among them two cases received volume intensity modulated radiotherapy (VMAT). A small number of cases were treated with tomotherapy. Clinical target volume (CTV) includes the PALN drainage region (T12 to L5 or matches the previous upper margin of the pelvic), a 5–15 mm setup margin for CTV was added to the planned target volume (PTV). Gross target volume (GTV) includes the enlarged PALNs or the surgery resection area, a 5 mm setup margin for GTV was added to the planned gross target volume (PGTV). In full consideration of the radiation tolerance of adjacent organs such as the pancreas, the dose given to the PALN region was 45–56.5 Gy with or without a PALN tumor boost up to 50–65 Gy, totally 23–30 fractions, with a fraction size of 1.5–2.4 Gy. And the frequency of radiotherapy is 5 times per week, once per day.

### Salvage treatment (chemotherapy)

In our study, 7 cases received concurrent platinum-containing chemotherapy during radiotherapy, including weekly cisplatin alone (carboplatin if cisplatin intolerant) and cisplatin + paclitaxel.

Besides, 13 cases received sequential chemoradiotherapy, including cisplatin + paclitaxel and cisplatin + gemcitabine.

In addition, 12 cases received chemotherapy alone, including cisplatin + paclitaxel.

### Salvage treatment (surgery ± adjuvant therapy)

In total, 15 cases received surgery, of whom 13 cases underwent radiotherapy after surgery, and among these 13 cases, 9 cases received combination of radiotherapy with chemotherapy (weekly cisplatin alone, cisplatin + paclitaxel, or cisplatin + gemcitabine).

### Follow-up

Patients were followed up at the first 1–2 months after the therapy, followed by every 3 months for 2 years, every 6 months for 2–5 years, and once a year thereafter. Gynecological examination, abdominal ultrasonography, CT scan, MRI, PET, or biopsy was performed in each follow-up. The recurrent disease was determined by gynecological imaging examination or biopsy. The evaluation of tumor response was based on Response Evaluation Criteria in Solid Tumours (RECIST) guideline (version 1.1). Complete response (CR): disappearance of all lesions; partial response (PR): at least a 30% decrease in the sum of diameters of lesions; progressive disease (PD): at least a 20% increase in the sum of diameters of target lesions; stable disease (SD): neither increase to qualify for PD nor decrease to qualify for PR [17]. The local control rate (LCR) was used to assess the treatment effect on the local recurrent tumor. Survival of the patients were assessed by overall survival (OS) and progression-free survival (PFS). OS referred to the duration from therapy to the last visit or death, and PFS indicated the duration from therapy to the last visit or recurrence.

### Statistical analysis

Statistical analysis was performed using the SPSS 17.0 statistical software (IBM, Armonk, NY, USA). Fisher’s exact test or Chi-square test was used to analyze categorical data. Univariate and multivariate Cox regression models were utilized to analyze the effects of different covariates on PFS and OS, which were expressed as hazard ratio (HR). The Kaplan–Meier method was used to evaluate survival curves and LCR, and log-rank test was used to analyze the difference in survival. P < 0.05 indicated significant difference.

## Results

### Patients’ characteristics

A total of 50 cervical cancer cases with isolated PALN recurrence were involved in the present study, and their features are listed in Table 1.

**Table 1:** Patients’ characteristics

Total	Number (%)
*At initial diagnosis*	
FIGO stage at initial diagnosis	
I	8 (16)
II	22 (44)
III	18 (36)
IV	2 (4)
Histology	
Squamous cell carcinoma	41 (82)
Adenocarcinoma	7 (14)
Adenosquamous carcinoma	2 (4)
Tumor size (cm)	
≤ 4	27 (54)
> 4	22 (44)
Unknown	1 (2)
Lower third of vaginal invasion	
No	44 (88)
Yes	5 (10)
Unknown	1 (2)
Pelvic lymph node	
Negative	12 (24)
Positive	38 (76)
Para-aortic lymph node	
Negative	48 (96)
Positive	2 (4)
Para-aortic radiotherapy	
No	45 (90)
Yes	5 (10)
Primary treatment	
Surgery ± adjuvant therapy	28 (56)
Radiotherapy only	6 (12)
Concurrent radiochemotherapy	16 (32)
SCC-Ag level (ng/mL)	
≤ 10	18 (36)
> 10	18 (36)
Unknown	14 (28)
CEA level (ng/mL)	
≤ 10	9 (18)
> 10	25 (50)
Unknown	16 (32)
CA199 level (ng/mL)	
≤ 37	30 (60)
> 37	5 (10)
Unknown	15 (30)
*At recurrence*	
Age at recurrence (years)	
≤ 57	31 (62)
> 57	19 (38)
SCC-Ag level (ng/mL)	
≤ 10	37 (74)
> 10	10 (20)
Unknown	3 (6)
CEA level (ng/mL)	
≤ 10	20 (40)
> 10	27 (54)
Unknown	3 (6)
CA199 level (ng/mL)	
≤ 37	39 (78)
> 37	8 (16)
Unknown	3 (6)
Detection of recurrence	
CT or MRI	26 (52)
PET/CT	24 (48)
Number of PALN recurrence	
One	13 (26)
Several	37 (74)
Sites of PALN recurrence	
Left lateral para-aortic	41 (58.6)
Aorto-caval	16 (22.9)
Right para-caval	10 (14.3)
Unknown	3 (4.3)
Highest level of PALN recurrence	
T12-L1	11 (22)
L2-L4	35 (70)
Unknown	4 (8)
Size of largest PALN recurrence (cm)	
≤ 1.5	18 (36)
> 1.5	29 (58)
Unknown	3 (6)
Clinical symptoms at recurrence	
None	35 (70)
Symptomatic	15 (30)
Time to PALN recurrence (months)	
≤ 12	22 (44)
> 12	28 (56)
Treatment after PLAN recurrence	
Surgery ± adjuvant therapy	15 (30)
Chemotherapy only	12 (24)
Concurrent chemoradiotherapy	7 (14)
Sequential chemoradiotherapy	13 (26)
Radiotherapy only	3 (6)
Tumor response	
CR	13 (26)
PR	28 (56)
SD	4 (8)
PD	5 (10)
Recurrence	
No	17 (34)
Yes	33 (66)
Second para-aortic recurrence	
No	34 (68)
Yes	16 (32)
Distant metastasis	
No	34 (68)
Yes	16 (32)
Death	
No	20 (40)
Yes	30 (60)

*PALN* Para-aortic lymph node, *CT* Computed tomography, *MRI* magnetic resonance, *PET* positron emission tomography, *SCC* squamous cell carcinoma, *FIGO* International Federation of Gynecology and Obstetrics, *CEA* Carcinoembryonic antigen, *CR* Complete response, *PR* Partial response, *SD* Stable disease, *PD* Progressive disease

At initial diagnosis, the mean age was 52 years old and the median age was 54 years old (range, 28–80 years); 38 (76%) cases had pelvic lymph node metastasis; only 2 (4%) cases had PALN metastasis.

The characteristics of these cases at recurrence are also summarized. As for treatment after PALN recurrence, 15 (30%) cases underwent surgery with or without adjuvant therapy, 13 (26%) cases received sequential radiochemotherapy, 12 (24%) cases underwent chemotherapy only; 7 (14%) cases received concurrent radiochemotherapy, and 3 (6%) cases underwent radiotherapy alone; and the disease control rate was 90%, CR was 13 (26%), PR was 28 (56%), SD was 4 (8%); recurrence was found in 33 (66%) cases, of whom, 16 cases had secondary para-aortic recurrence and 16 cases had distant metastasis; regrettably, 30 (60%) cases died.

Surgical details for cases with isolated PALN recurrence are shown in Table 2. Among the 15 cases who underwent paraaortic lymphadenectomy, the repair of vena cava injury was found in 5 cases, pelvic lymphadenectomy in 1, duodenal tumor resection in 1, duodenorrhaphy in 1, partial resection of psoas major muscle in 1, placement of ureteral stents in 1, and insertion of a small intestinal decompression tube during the surgery in 1.

**Table 2:** Characteristics of surgery for PLAN recurrence

	Number (%)
Surgical approach	
Laparotomy	10 (66.7)
Laparoscopy	5 (33.3)
Operative time, min (mean)	36.36 ± 9.224
Intraoperative bleeding, ml (mean)	180.71 ± 263.365
Surgical procedures	
Paraaortic lymphadenectomy	15 (100)
Repair of vena cava injury	5 (33.3)
Pelvic lymphadenectomy	1 (6.7)
Duodenal tumor resection	1 (6.7)
Duodenorrhaphy	1 (6.7)
Partial psoas major muscle resection	1 (6.7)
Ureteral stents placement	1 (6.7)
Small intestinal decompression tube insertion	1 (6.7)
Operative complications	
None	8 (53.3)
Intestial injury	1 (6.7)
Vascular injury	5 (33.3)
Ureteral injury	1 (6.7)
Postoperative complications	
None	7 (46.7)
Lymphocyst	4 (26.7)
Infection	1 (6.7)
Ileus	2 (13.3)
Chylous fistula	1 (6.7)
Length of hospital stays, day (mean)	11.07 ± 4.763
Residual disease	
None	13 (86.7)
> 0	2 (13.3)

*PALN* para-aortic lymph node

After surgery, 4 (26.7%) cases had lymphocyst, 1 (6.7%) had infection, 2 (13.3) had ileus, and 1 (6.7%) had chylous fistula. Up to 86.7% of cases had no residual disease.

### LCR

The results of univariate analysis of LCR are presented in Table 3. Kaplan–Meier survival analysis showed that CEA level > 10 ng/mL at both initial diagnosis (P = 0.047) and recurrence (P = 0.012), and PET/CT used to detect the recurrence (P = 0.006) were significantly associated with 3-year LCR.

**Table 3:** Univariate analyses of local control rate

	Number	3-year (%)	P value
*At initial diagnosis*			
FIGO stage at initial diagnosis			0.901
I	8	75	
II	22	57.4	
III	18	69.3	
IV	2	50	
Histology			0.381
Squamous cell carcinoma	41	59.7	
Adenocarcinoma	7	66.7	
Adenosquamous carcinoma	2	100	
Tumor size (cm)			0.060
≤ 4	27	76.7	
> 4	22	48.3	
Lower third of vaginal invasion			0.150
No	44	64.9	
Yes	5	40	
Pelvic lymph node			0.234
Negative	12	42.8	
Positive	38	69.5	
Para-aortic lymph node			0.289
Negative	48	64.6	
Positive	2	50	
Para-aortic radiotherapy			0.146
No	45	67.5	
Yes	5	40	
Primary treatment			0.966
Surgery ± adjuvant therapy	28	65.6	
Radiotherapy alone	6	41.7	
Concurrent radiochemotherapy	16	65.3	
SCC-Ag level (ng/mL)			0.164
≤ 10	18	62.4	
> 10	18	52.6	
CEA level (ng/mL)			**0.047**
≤ 10	9	80	
> 10	25	43.7	
CA199 level (ng/mL)			0.528
≤ 37	30	53.8	
> 37	5	66.7	
*At recurrence*			
Age at recurrence (years)			0.130
≤ 57	31	56.8	
> 57	19	73.7	
SCC-Ag level (ng/mL)			0.792
≤ 10	37	60.5	
> 10	10	64	
CEA level (ng/mL)			**0.012**
≤ 10	20	84.2	
> 10	27	44.6	
CA199 level (ng/mL)			0.128
≤ 37	39	66.8	
> 37	8	29.2	
Detection of recurrence			**0.006**
CT or MRI	26	47.5	
PET/CT	24	82	
Number of PALN recurrence			0.091
One	13	81.8	
Several	37	54.7	
Highest level of PALN recurrence			0.774
T12-L1	11	65.5	
L2-L4	35	64.4	
Size of largest PALN recurrence (cm)			0.228
≤ 1.5	18	55	
> 1.5	29	65.8	
Clinical symptoms at recurrence			0.887
None	35	63	
Symptomatic	15	71.8	
Time to PALN recurrence (months)			0.672
≤ 12	22	71.4	
> 12	28	56.8	
Treatment after PLAN recurrence			0.086
Surgery ± adjuvant therapy	15	84	
Chemotherapy only	12	28.1	
Concurrent chemoradiotherapy	7	64.3	
Sequential chemoradiotherapy	13	62.5	
Radiotherapy only	3	66.7	

*PALN* Para-aortic lymph node, *CT* Computed tomography, *MRI* magnetic resonance, *PET* positron emission tomography, *SCC* squamous cell carcinoma, *FIGO* International Federation of Gynecology and Obstetrics, *CEA* Carcinoembryonic antigen

Bold numbers represent P < 0.05

**Fig. 1:**
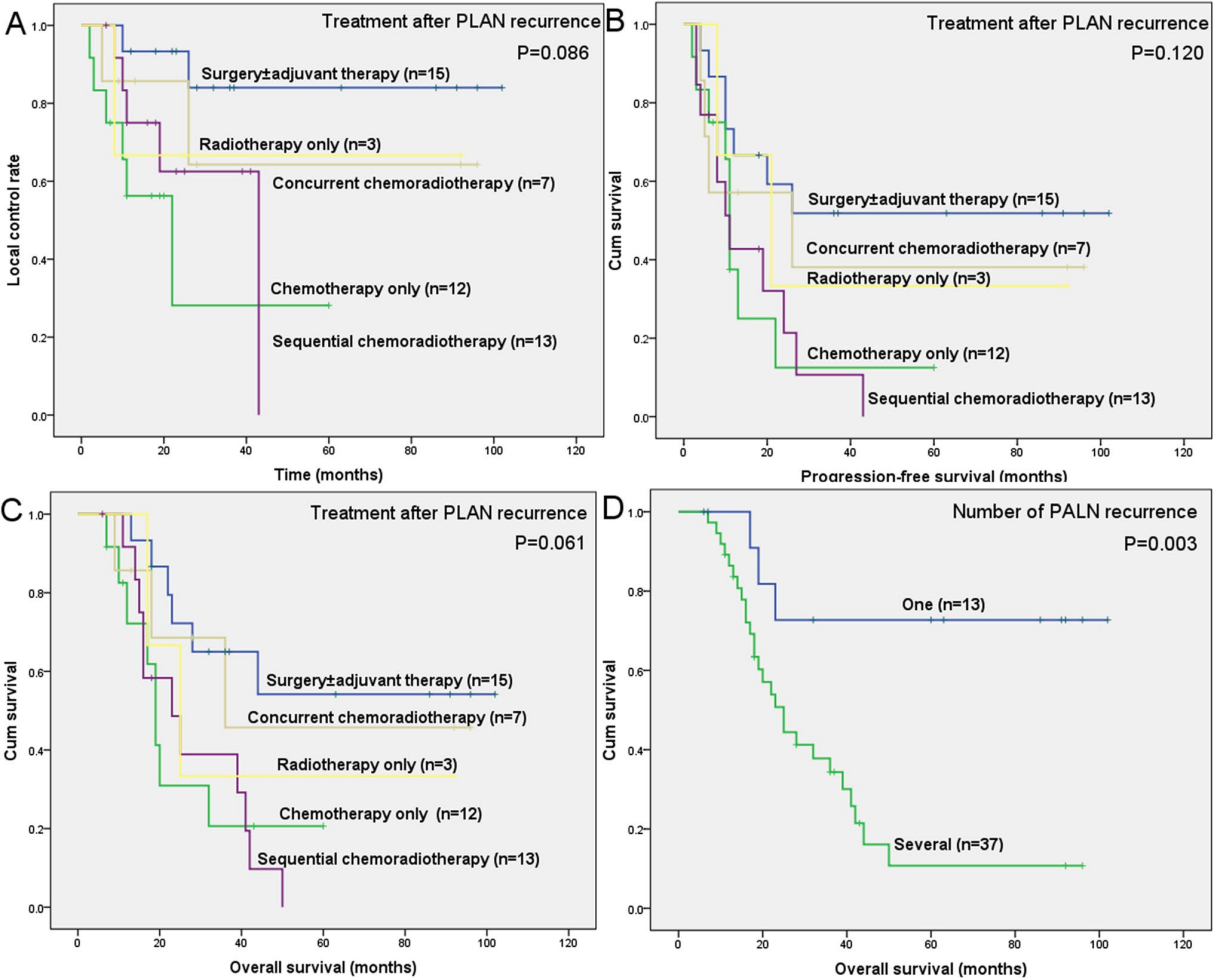
**A** Comparison of local control rate in patients with isolated recurrent PLAN based on different treatments after recurrence; **B** Comparison of progression-free survival curve in patients with isolated recurrent PLAN based on different treatments after recurrence; **C** Comparison of overall survival curve in patients with isolated recurrent PLAN based on different treatments after recurrence; **D** Comparison of overall survival curve in patients with isolated recurrent PLAN based on the number of PLAN recurrence

Univariate Cox regression analysis suggested that the 3-year LCR of chemotherapy alone was significantly lower than that of surgery with or without adjuvant therapy (28.1% vs. 84%), and the risk of secondary para-aortic recurrence increased by 7.828 times (P = 0.014). Using Kaplan–Meier analysis, the LCR curves of the different treatments after PALN recurrence were plotted and presented in Fig. 1.

### Survival

The 3-year OS of the whole cases was 43.9% and the median OS was 28 months.

#### Univariate and multivariate Cox regression analyses of survival after recurrence (Table 4)

**Table 4:** Prognostic factors for survival after recurrence in univariate and multivariate analyses

	Progression-free survival				Overall survival			
	Univariate		Multivariate		Univariate		Multivariate	
	HR (95%CI)	P value	HR (95%CI)	P value	HR (95%CI)	P value	HR (95%CI)	P value
*At initial diagnosis*								
FIGO stage at initial diagnosis								
I	1				1			
II	1.513 (0.497–4.605)	0.466			0.976 (0.312–3.048)	0.966		
III	2.185 (0.715–6.671)	0.170			1.925 (0.626–5.919)	0.253		
IV	1.014 (0.113–9.101)	0.990			0.816 (0.090–7.361)	0.856		
Histology								
Squamous cell carcinoma	1				1			
Adenocarcinoma	0.331 (0.079–1.388)	0.131			0.469 (0.111–1.973)	0.301		
Adenosquamous carcinoma	0.428 (0.058–3.150)	0.405			0.472 (0.064–3.479)	0.461		
Tumor size (cm)		**0.010**		0.521		0.288		
≤ 4	1		1		1			
> 4	2.563 (1.254–5.240)		1.892 (0.270–13.268)		1.49 (0.714–3.109)			
Lower third of vaginal invasion		0.863				0.948		
No	1				1			
Yes	0.900 (0.273–2.970)				0.961 (0.289–3.197)			
Pelvic lymph node		0.851				0.112		
Negative	1				1			
Positive	0.926 (0.416–2.063)				0.513 (0.225–1.168)			
Para-aortic lymph node		**0.003**		**0.005**		0.140		
Negative	1		1		1			
Positive	12.182 (2.326–63.811)		20.951 (2.545–172.500)		2.992 (0.697–12.840)			
Para-aortic radiotherapy		0.180				0.475		
No	1				1			
Yes	1.922 (0.739–5.001)				1.472 (0.510–4.250)			
Primary treatment								
Surgery ± adjuvant therapy	1				1			
Radiotherapy alone	1.849 (0.671–5.097)	0.235	4.680 (0.886–24.730)	0.069	1.652 (0.590–4.620)	0.339		
Concurrent radiochemotherapy	2.190 (1.034–4.636)	**0.041**	2.010 (0.581–6.955)	0.270	1.893 (0.854–4.197)	0.116		
SCC-Ag level (ng/mL)		**0.040**		**0.006**		0.209		
≤ 10	1		1		1			
> 10	2.357 (1.039–5.343)		7.027 (1.766–27.952)		1.746 (0.732–4.163)			
CEA level (ng/mL)		0.051				0.080		
≤ 10	1				1			
> 10	3.377 (0.994–11.469)				3.016 (0.876–10.385)			
CA199 level (ng/mL)		0.260				0.841		
≤ 37	1				1			
> 37	1.891 (0.625–5.722)				1.135 (0.331–3.888)			
*At recurrence*								
Age at recurrence (years)		0.409				0.541		
≤ 57	1				1			
> 57	0.741 (0.363–1.512)				0.796 (0.382–1.656)			
SCC-Ag level (ng/mL)		0.878				0.355		
≤ 10	1				1			
> 10	0.933 (0.383–2.271)				0.607 (0.210–1.749)			
CEA level (ng/mL)		**0.026**		**0.005**		0.105		
≤ 10	1		1		1			
> 10	2.429 (1.112–5.307)		11.197 (2.038–61.512)		1.934 (0.871–4.296)			
CA199 level (ng/mL)		0.819				0.917		
≤ 37	1				1			
> 37	0.884 (0.307–2.546)				1.058 (0.366–3.053)			
Detection of recurrence		0.253				0.283		
CT or MRI	1				1			
PET/CT	0.667 (0.334–1.335)				0.672 (0.325–1.388)			
Number of PALN recurrence		**0.018**		**0.009**		**0.007**		**0.014**
One	1		1		1		1	
Several	3.600 (1.241–10.443)		20.818 (2.111–205.310)		5.245 (1.557–17.667)		5.386 (1.410–20.570)	
Highest level of PALN recurrence		0.557				0.946		
T12-L1	1				1			
L2-L4	0.784 (0.347–1.768)				1.032 (0.416–2.562)			
Size of largest PALN recurrence (cm)		0.519				0.720		
≤ 1.5	1				1			
> 1.5	0.792 (0.390–1.609)				0.874 (0.417–1.830)			
Clinical symptoms at recurrence		0.740				0.458		
None	1				1			
Symptomatic	0.873 (0.392–1.944)				1.362 (0.602–3.081)			
Time to PALN recurrence (months)		0.601				0.594		
≤ 12	1				1			
> 12	0.833 (0.419–1.654)				0.823 (0.401–1.687)			
Treatment after PLAN recurrence								
Surgery ± adjuvant therapy	1		1		1		1	
Chemotherapy only	2.866 (1.046–7.853)	**0.041**	0.101 (0.008–1.319)	0.080	3.418 (1.176–9.935)	**0.024**	2.446 (0.828–7.223)	0.105
Concurrent chemoradiotherapy	1.548 (0.453–5.291)	0.486	0.170 (0.031–0.922)	**0.040**	1.364 (0.341–5.458)	0.660	0.649 (0.153–2.743)	0.556
Sequential chemoradiotherapy	3.045 (1.168–7.934)	**0.023**	0.278 (0.031–2.502)	0.253	3.501 (1.284–9.543)	**0.014**	1.745 (0.609–5.004)	0.300
Radiotherapy only	1.561 (0.324–7.527)	0.579	0.173 (0.024–1.263)	0.084	1.884 (0.380–9.345)	0.438	2.232 (0.446–11.180)	0.329

*PALN* Para-aortic lymph node, *CT* Computed tomography, *MRI* magnetic resonance, *PET* positron emission tomography, *SCC* squamous cell carcinoma, *FIGO* International Federation of Gynecology and Obstetrics, *CEA* Carcinoembryonic antigen, *HR* hazard ratio, *CI* confidence intervals

Bold numbers represent P < 0.05

Univariate Cox regression analysis suggested that 7 factors were significantly associated with PFS, including tumor size (> 4 cm) (HR = 2.563, P = 0.010), para-aortic lymph node (positive) (HR = 12.182, P = 0.003), primary treatment (CCRT vs. surgery ± adjuvant therapy) (HR = 2.190, P = 0.041), SCC-Ag level (> 10 ng/mL) (HR = 2.357, P = 0.040) at initial diagnosis, CEA level (> 10 ng/mL) (HR = 2.429, P = 0.026), the number of PALN recurrences (several) at recurrence (HR = 3.600, P = 0.018), and treatment after PALN recurrence (CT vs. surgery ± adjuvant therapy, HR = 2.866, P = 0.041; SCRT vs. surgery ± adjuvant therapy, HR = 3.045, P = 0.023).

All variables with P < 0.05 in the univariate analysis were analyzed by the multivariate Cox regression analysis. The results showed that para-aortic lymph node (positive), SCC-Ag level (> 10 ng/mL) at initial diagnosis, CEA level (> 10 ng/mL), and number of PALN recurrences (several) at recurrence were independent prognostic factors. In addition, compared with surgery ± adjuvant therapy, CCRT after PALN recurrence could reduce the risk of the second recurrence.

In the univariate Cox regression analysis of the OS, only the number of PALN recurrences (several) at recurrence (HR = 5.245, P = 0.007) and treatment after PALN recurrence (CT vs. surgery ± adjuvant therapy, HR = 3.418, P = 0.024; SCRT vs. surgery ± adjuvant therapy, HR = 3.501, P = 0.014) were significant factors.

And in the multivariate Cox regression analysis of the OS, only the number of PALN recurrences (several) at recurrence (HR = 5.386, P = 0.014) was an independent prognostic factor.

### Summary of outcomes of isolated PALN recurrence after different salvage treatments

Literature review of cervical cancer cases with isolated PALN recurrence after different salvage treatments are summarized in Table S1. The salvage treatments included radiotherapy (RT), chemotherapy (CT), and CCRT. Surgery was performed in few studies.

In our study, 3-year OS for surgery ± adjuvant therapy, CCRT, SCRT, RT, and CT was 65%, 45.7%, 38.9%, 33.3%, and 20.6%, respectively (Table 5). And the survival curves of PFS and OS related to different salvage treatments are displayed in Fig. 1.

**Table 5:** Outcomes for isolated PALN recurrence after different salvage treatment

Number	Salvage treatment	Outcome
50		3-y OS: 43.9% Median OS: 28 months
	CT:24%	(CT-3-y OS: 20.6% Median OS: 19 months)
	RT:6%	(RT-3-y OS: 33.3% Median OS: 25 months)
	CCRT:14%	(CCRT-3-y OS: 45.7% Median OS: 36 months)
	SCRT:26%	(SCRT-3-y OS: 38.9% Median OS: 23 months)
	Surgery ± adjuvant therapy:30%	(Surgery ± adjuvant therapy-3-y OS: 65%)

*PALN* Para-aortic lymph node, *OS* Overall survival, *CT* Chemotherapy, *RT* Radiotherapy, *CCRT* Concurrent chemoradiotherapy, *SCRT* Sequential chemoradiotherapy

## Discussion

Niibe et al. [16] evaluated 3,137 cases with cervical cancer of stage Ia to Iva, in which the rate of isolated PALN recurrence was 67 (2.1%), and the mean time between primary treatment and PALN recurrence was 20 months. SCC-Ag level or CEA level ≥ 10 ng/mL, positive pelvic lymph nodes upon initial diagnosis, and the overexpression of cyclooxygenase-2 in the primitive tumor tissues were risk factors of PALN relapse [18, 19].

A study identified 92 PALN recurrence in 35 cervical cancer cases, among which 46.8% were at left lateral para-aortic, 38.0% were at aorto-caval, and 15.2% were at right para-caval areas [20]. As demonstrated in our study, the left lateral para-aortic recurrence occurred most frequently (58.6%).

The prognosis was always poor in these patients [21]. Several studies have assessed factors associated with the prognosis of cervical cancer cases with isolated PALN recurrence.

In a study conducted by Kubota et al. [4], SCC-Ag level ≥ 1.5 ng/mL, the number of PALN recurrences, and treatment for PALN recurrence were associated with LCR, in which the 3-year LCR for CCRT and surgery was both 100%, the 3-year LCR for RT was 55.5%, and the 3-year LCR for CT was just 33.6% (P = 0.028). In our study, CEA level > 10 ng/mL and PET/CT used to detect the recurrence were associated with 3-year LCR, and the 3-year LCR of CT alone was significantly lower than that of surgery ± adjuvant therapy (28.1% vs. 84%). However, the number of PALN recurrences and SCC-Ag level were not the significant factors on LCR, the reason may be SCC-Ag level data is incomplete, fewer patients just have one lymph node recurrence. A larger sample and more complete data are needed in future research.

The highest level of PALN recurrence, clinical symptoms at recurrence, and time to PALN recurrence were reported to be related to survival in cervical cancer cases with isolated PALN recurrence. In a study of 65 cervical cancer cases with PALN recurrence, the highest level of PALN recurrence (≥ L1) predicted a poor survival and a high rate of distant metastases, and the clinical symptoms of leg edema and/or back pain at recurrence were associated with worse OS [11]. According to the research conducted by Cho et al. [12], Jeon et al. [9], and Kim et al. [6], time to PALN recurrence after initial therapy for more than 12, 18, and 24 months was associated with a better survival. However, survival rates in our study were not significantly affected by these factors. This may be because most of the patients enrolled in our study had no clinical symptoms and the highest level of PALN recurrence was at the L2–L4.

Several studies did not find significant effects of positive para-aortic lymph node at initial diagnosis on the prognosis of cervical cancer cases with PALN recurrence. However, in the present research, positive para-aortic lymph nodes at initial diagnosis were found as an independent factor related to worse PFS.

In addition, a number of scholars have investigated the effects of SCC-Ag and CEA levels on the prognosis of cases with PALN recurrence. Jeon et al. [9] suggested that SCC-Ag level (≤ 8 ng/dL) at the time of recurrence was associated with a good prognosis.

Huang et al. [15] found that CEA level ≥ 10 ng/mL was a negative factor for both PFS and OS, and more aggressive therapy might be advisable for these cases. And it was also observed in our research, SCC-Ag level (> 10 ng/mL) and CEA level (> 10 ng/mL) were related to the PFS.

In a study conducted by Kubota et al. [4], the number of PALN recurrences was significantly associated with PFS. In our study, the number of PALN recurrences was associated with PFS and OS.

CCRT had been proven to be an effective treatment for cervical cancer cases with PALN recurrence [6–10]. In a study performed by Chou et al. [10], 26 cervical cancer cases with isolated PALN recurrence were analyzed, and the treatment included CT, RT, and CCRT. It was found that 7 of 26 cases were alive, and all 7 survivors had undergone CCRT. Similarly, our results also demonstrated that CCRT after PALN recurrence could reduce the risk of the second recurrence.

According to clinical experience, cases who underwent satisfied surgery for recurrent PALN with or without adjuvant therapy after surgery might have a good prognosis. However, few studies have assessed surgery ± adjuvant therapy and survival of cases who suffered from isolated PALN recurrence. In our study, the univariate analysis showed that compared with surgery ± adjuvant therapy, chemotherapy alone or sequential chemoradiotherapy was associated with worse PFS or OS, while the risk was not significant in the multivariate Cox regression analysis. In a study conducted by Kubota et al. [4], for cases with isolated PALN recurrence, 3-year OS for cases undergoing surgery was high to 66.7%, which was secondary only to CCRT, while Cho et al. [12] found that the outcome was worse in cases who received surgery ± other modalities than those receiving RT, CT or CCRT. In our research, the surgical outcome was promising, and 3-year OS of cases after surgery ± adjuvant therapy was the highest (65%), followed by CCRT (45.7%), SCRT (38.9%), RT (33.3%), and CT (20.6%). It may be related to satisfactory tumor reduction and localized lesions in cases with surgery, in which up to 86.7% of cases (13/15) had no residual disease, and nearly half of the cases had only one lesion.

There are some limitations in this study. First, the number of cases was limited and the study was retrospective, which were the limitations in obtaining results with high confidence. Other studies with larger sample from multi centers or prospective studies were required to increase the credibility and reference value of the results. Second, a large number of factors have been considered in the survival analysis, which may result in a statistical significance by chance.

## Conclusions

In cervical cancer cases with isolated PALN recurrence, CEA level ≤ 10 ng/mL may predict better LCR, and PET/CT appeared more reliable to exclude occult metastases and to improve LCR. Positive PALN, SCC-Ag level > 10 ng/mL at initial diagnosis, and CEA level > 10 ng/mL, the number of PALN metastases (several) at the time of recurrence predicted worse survival. Additionally, among these patients, CCRT or surgery ± adjuvant therapy may be preferred as the treatment after the recurrence. However, additional larger sample size from multicenter or prospective studies are required to verify our findings.

### Supplementary Information

Below is the link to the electronic supplementary material.

Supplementary file 1 (DOCX 19 KB)

## Data Availability

The related data were available from the corresponding author upon reasonable request.

## Abbreviations

PALN: Para-aortic lymph node
CT: Computed tomography
MRI: Magnetic resonance
PET: Positron emission tomography
SCC: Squamous cell carcinoma
FIGO: International Federation of Gynecology and Obstetrics
CEA: Carcinoembryonic antigen
IMRT: Intensity-modulated radiation therapy
VMAT: Volume intensity modulated radiotherapy
CTV: Clinical target volume
PTV: Planned target volume
GTV: Gross target volume
PGTV: Planned gross target volume
RECIST: Response Evaluation Criteria in Solid Tumours
CR: Complete response
PR: Partial response
SD: Stable disease
PD: Progressive disease
HR: Hazard ratio
CI: Confidence intervals
PFS: Progression-free survival
OS: Overall survival
CCRT: Concurrent chemoradiotherapy
SCRT: Sequential chemoradiotherapy
SPSS: Statistical Package for Social Sciences
