# Supplementary material for: The impact of different clinicopathologic factors and salvage therapies on cervical cancer patients with isolated para-aortic lymph node recurrence
Source: Discov Oncol. 2024 Mar 1;15:54. doi: 10.1007/s12672-023-00825-w (PMC10907327; doi:10.1007/s12672-023-00825-w)
Supplement: Supplementary file 1 — (DOCX 19 KB) [file 12672_2023_825_MOESM1_ESM.docx]

| **Table S1** Literature review of cervical cancer cases with isolated PALN recurrence after different salvage treatments | | | | |
| --- | --- | --- | --- | --- |
| **Author** | **Year** | **Number** | **Salvage treatment** | **Outcome** |
| Grigsby et al. [5] | 1994 | 20 | RT: 100% | Median OS: 8.7 months |
| Chou et al. [10] | 2001 | 26 | RT: 3.8% | 5-y OS: 30.8% |
|  |  |  | CCRT: 53.8% | (CCRT-5-y OS: 51.2%) |
|  |  |  | CT:15.4% |  |
|  |  |  | None:27.0% |  |
| Kim et al. [6] | 2003 | 12 | CCRT: 100% | 3-y OS: 19% |
|  |  |  |  | (Median OS: 21 months) |
| Singh et al. [8] | 2005 | 14 | CT:21.4% | 5-y OS: 50% |
|  |  |  | RT:21.4% | (Asymptomatic patients, CCRT-5-y OS: 100%) |
|  |  |  | CCRT:50% |  |
|  |  |  | None:7.1% |  |
| Niibe et al. [3] | 2006 | 84 | RT: 61.9% | 3-y OS: 49.5% 5-y OS: 31.3% |
|  |  |  | CCRT: 38.1% |  |
| Cho et al. [12] | 2019 | 32 | CT | (CT-3-y OS: 50%) |
|  |  |  | RT | (RT-3-y OS: 50%) |
|  |  |  | CCRT | (CCRT-3-y OS: 76.4%) |
|  |  |  | Surgery±other modalities | (Surgery±other modalities-3-y OS: 33.3%) |
| Chen et al. [11] | 2019 | 46 | CT:15.4% | Median OS: 27.7 months 5-y OS: 33.9% |
|  |  |  | RT:16.9% | (CT-3-y OS: 40%) |
|  |  |  | CCRT:38.5% | (RT-3-y OS: 44.7%) |
|  |  |  | None:29.2% | (CCRT-3-y OS: 48.4%) |
| Kubota et al. [4] | 2019 | 50 |  | 3-y OS: 47% |
|  |  |  | CT:34% | (CT-3-y OS: 48.8%) |
|  |  |  | RT:36% | (RT-3-y OS: 41.3%) |
|  |  |  | CCRT:14% | (CCRT-3-y OS: 85.7%) |
|  |  |  | Surgery:6% | (Surgery-3-y OS: 66.7%) |
|  |  |  | Best supportive care:10% | (Best supportive care-3-y OS: 0) |
| *PALN, Para-aortic lymph node; OS, Overall survival; CT, Chemotherapy; RT, Radiotherapy; CCRT, Concurrent chemoradiotherapy; SCRT, Sequential chemoradiotherapy* | | | | |
